# Self-Sacrifice in a Distressful and Threatening Environment: The Consequences of the COVID-19 Crisis in Intensifying Workplace Violence

**DOI:** 10.3389/fpsyt.2022.848059

**Published:** 2022-05-17

**Authors:** Zahra Ebrahimi Rigi, Parvin Mangolian Shahrbabaki, Fazlollah Ahmadi, Ali Ravari

**Affiliations:** ^1^Nursing Research Center, Kerman University of Medical Sciences, Kerman, Iran; ^2^Nursing Research Center, Department of Critical Care Nursing, Razi Faculty of Nursing and Midwifery, Kerman University of Medical Sciences, Kerman, Iran; ^3^Department of Nursing, Faculty of Medical Sciences, Tarbiat Modares University, Tehran, Iran; ^4^Geriatric Care Research Center, School of Nursing and Midwifery, Rafsanjan University of Medical Sciences, Rafsanjan, Iran

**Keywords:** workplace violence, nurses, COVID-19, crisis, self-sacrifice

## Abstract

**Background:**

The stress and mental pressure resulting from the challenges posed by the COVID-19 crisis exacerbated occupational stressors such as workplace violence against nurses even though nurses were endangering their lives to provide high-quality care and reduce patients’ suffering. Therefore, the present study aimed to explain Iranian nurses’ experiences of workplace violence during the COVID-19 crisis.

**Materials and Methods:**

This study was conducted using a qualitative approach. Twenty-five semi-structured interviews were conducted with nurses who had experienced workplace violence at COVID-19 referral centers in Kerman during the COVID-19 crisis. Conventional content analysis was used to analyze the data, and the research was reported *via* the COREQ checklist.

**Results:**

Analysis of the findings led to one main category, “nurses’ self-sacrifice in a distressful and threatening environment,” and four subcategories, which included “omitting entertainment and fun activities,” “having challenging duties in unsafe conditions,” “receiving insufficient support,” and “nurses’ toleration of disrespect.”

**Conclusion:**

Crises can exacerbate workplace violence toward nurses. Thus, it is necessary to design educational programs and prevention strategies to manage the destructive psychological and occupational impact of the crises on nurses. Nurses should receive training in crisis management to cope with the intensified aggressive behavior of managers, colleagues, patients, and patient companions during the crisis. Policy-makers must be prepared to deal with crises, and they should take measures to improve nurses’ mental health and quality of care.

## Introduction

The COVID-19 pandemic has been the most significant global health crisis in recent decades (1). Beyond its health consequences, the pandemic has significantly affected health systems (2). Nurses are valuable assets of the health system who work in the frontline of health care provision during the pandemic; this means that they are the personnel most affected by it (2). The nursing profession is inseparable from values such as “empathy, love, and self-sacrifice,” so a culture of self-sacrifice has become an integral element of this occupation (3). In spite of the harsh conditions, nurses make sacrifices, provide for patients’ needs, and disregard their own needs and wellness, spending all their power on providing quality care and fulfilling patients’ needs (4). From the beginning of the COVID-19 crisis, nurses have faced threats, danger, and uncertainty caused by infection, fear of transmitting the virus (5), death anxiety (6), and the necessity of quick adaptation to rapid changes in care procedures (7). These concerns and challenges impose significant mental pressure on nurses (7). The continuation and exacerbation of stressful conditions can aggravate other occupational stressors such as workplace violence (8). Workplace violence is an occupational psychosocial risk factor; psychosocial risk factors are classified into two categories: those of an individual and malicious nature (violence in the workplace including harassment, bullying) and those of a culpable and collective nature (burnout syndrome and work-related stress), which can aggravate each other and damage the worker’s health (9). Workplace violence refers to any incident or situation in which a person is subjected to abuse, harassment, threat, or assault at their workplace or in the circumstances related to their work (10). Nurses are the most common victims of workplace violence (11). The growth of workplace violence is a serious threat to nurses’ mental health (12). It leads to decreased resistance, clinical burnout, and the inability to provide high-quality and professional care in nurses (13). A recent systematic review reported that the prevalence of workplace violence against nurses was 67.5–90.4% (14). A study in Iran found that violence in nurses’ workplaces is committed by their colleagues and superiors or patients and their relatives. Also, depending on various complicated factors at individual and organizational levels, this violence may be caused by unmet expectations of patients or their relatives, inefficient administrative management, and improper professional communication (15). Most recent descriptive and analytical studies conducted on the effects of the COVID-19 crisis on nurses have focused on nurses’ work quality (16), mental health (7), self-efficacy, and psychological disorders, such as anxiety and depression (17), emotional responses to fear and stress (18), occupational dissatisfaction (19), and job burnout (20). Qualitative studies have also focused on similar issues (21) and also on nurses’ experiences with COVID-19 patients (22).

Critical circumstances such as the COVID-19 crisis cause high levels of stress and anxiety (23). For example, the explosion in the number of new COVID-19 infections and the high mortality rate have caused public fear and anxiety. Plus, people were asked to strictly avoid familial gatherings and trips, which led to increased stress. Hence, there was increased friction between the management and the medical staff, including nurses, which added much psychological pressure to the already increased workload of nurses (1). Moreover, nurses’ tireless efforts in such difficult conditions were sometimes unappreciated (24). The social and cultural context affected these complex and multidimensional phenomena (2). Because this phenomenon has a subjective nature and cannot be measured by quantitative methods, it seems that qualitative research is required to explain how the COVID-19 crisis has affected the prevalence of workplace violence. Therefore, qualitative research can help achieve a correct understanding of nurses’ experiences and create opportunities for discovering the problems and dealing with them (15).

Workplace violence is a topic of great importance to healthcare personnel, especially to nurses. The growth in workplace violence against nurses has become a severe issue that can be aggravated even further in critical circumstances. Numerous studies have been conducted on the effects of the COVID-19 crisis on nurses (2, 16, 23); however, there is little information on workplace violence during the COVID-19 crisis. Also, we did not find any study on the impact of the COVID-19 crisis on workplace violence against nurses in Iran. Therefore, the present study aimed to explain Iranian nurses’ experiences of workplace violence during the COVID-19 crisis.

## Materials and Methods

### Study Design

This qualitative study applied the conventional content analysis method with a descriptive-explorative approach (25). A qualitative study is a critical tool for studying emotions, perceptions, and knowledge about the complexities of human reactions, which cannot be obtained *via* quantitative research. Content analysis is a systematic coding and categorizing method used to understand, analyze, and conceptualize the underlying concepts of qualitative data (26).

### Sample and Setting

Due to the qualitative nature of the data, the study settings had to be real, so this study’s setting included public and semi-private referral hospitals of Kerman. A total of 25 individuals (including 18 female nurses and 7 male nurses) participated in this study (Table 1). Nurses who had experienced workplace violence during the COVID-19 crisis were selected from referral centers in Kerman. Interviews were done in prearranged meetings at the participants’ convenience. Participants were selected for purposeful sampling from May 2020 to July 2022, and interviews continued until data saturation was reached when no new concepts were extracted from new data (26). The present study reached saturation after interviewing 22 participants, but three additional interviews were conducted to confirm data saturation. The criteria for selecting the participants included having experienced workplace violence in the COVID-19 crisis and willingness to recount these experiences. The participants who had a history of mental illness or those taking sedatives or antianxiety or antidepressant medications were excluded from the study. Participants were selected based on the maximum variation principle, with different ages, genders, work experience, work experience in COVID-19 wards, and working shifts. Personal information such as marital status, academic degree, and position was recorded for a broader range of information. In-depth interviews were performed individually and face-to-face. Each interview lasted from 45 to 90 min.

**TABLE 1:** Characteristics of participants (*N* = 25).

Participants	Age (years)	Gender/marital status	Experience in nursing (years)	Duration of working at corona wards
1	24	Female/single	6 months	15-day rotation
2	36	Female/married	11	From the beginning of the coronavirus outbreak
3	43	Female/married	15	Admission and care of patients
4	23	Male/single	3	Admission and care of patients
5	32	Female/married	6	From the beginning of the coronavirus outbreak
6	32	Male/married	8	From the beginning of the coronavirus outbreak
7	34	Female/single	10	From the beginning of the coronavirus outbreak
8	32	Female/single	7	Auxiliary force
9	24	Male/single	3	From the beginning of the coronavirus outbreak
10	32	Female/married	3	From the beginning of the coronavirus outbreak
11	26	Female/single	4	From the beginning of the coronavirus outbreak
12	25	Male/single	3	Auxiliary force
13	38	Female/married	11	From the beginning of the coronavirus outbreak
14	28	Male/single	5	Auxiliary force
15	36	Female/married	9	Auxiliary force
16	27	Female/married	3	From the beginning of the coronavirus outbreak
17	26	Female/married	4	Admission and care of patients
18	37	Female/single	9	Auxiliary force
19	24	Male/single	6 months	15-day rotation
20	32	Female/single	9	Admission and care of patients
21	25	Female/widow	4	Admission and care of patients
22	44	Male/married	18	From the beginning of the coronavirus outbreak
23	43	Female/single	15	Auxiliary force
24	32	Female/single	11	Admission and care of patients
25	35	Female/single	9	Admission and care of patients

### Data Collection Procedure

Open-ended, semi-structured, in-depth interviews were conducted for data collection. Initially, some prepared questions were asked to familiarize the researcher with the participant and produce a pleasant atmosphere. Then the interview was guided toward the aim of the study. Also, field notes were taken for collecting data. The primary question asked from the participants was, “Please talk about your experience of workplace violence during the COVID-19 crisis.” Based on the participant’s answers, exploratory questions, like “Could you explain more?” or “Would you give an example?” were asked to obtain more in-depth information. All interviews were performed by the first author, who had adequate experience in conducting interviews. After obtaining informed written consent for data recording and ensuring the participants of the confidentiality of data, the interview started by giving some information, including study aims and collection method, to the participants. The MAXQDA 10 was used to code and extract categories and themes.

### Ethical Considerations

The Ethics Committee of Kerman University of Medical Sciences approved all the procedures used in the study (code: IR.KMU.REC.1398.174). This study was conducted following the ethical guidelines outlined in the Declaration of Helsinki. The study aims and collection method were clearly explained when recruiting participants, and written informed consent was obtained. Participants were informed of the recording of the interviews. The place and time of the interviews were chosen according to the participants’ preferences. The participants were assured of the confidentiality of all the gathered data. All audio files were stored securely and deleted after the final report. Participants were able to withdraw from the study at any time. In addition, participants were requested to contact us if they had any questions.

### Data Analysis

Data analysis was done using content analysis and based on Graneheim and Lundman’s five steps (27). The researcher transcribed the recordings immediately after conducting the interviews. In the second step, the full texts of the interviews were read repeatedly to gain a general understanding of their content. In the third step, all the transcripts of the interviews were read to determine the meaning units relevant to the aim of the study; the meaning units were summarized, maintaining their content, and labeled with suitable codes. In the fourth step, the researchers created subcategories based on similarities and differences of codes. Table 2 presents some examples. The first and second authors separately coded one interview to evaluate agreement on the codes, and 84% agreement was observed. In the fifth step, after identifying latent content, subcategories were placed in the main categories, which were conceptually more comprehensive and abstract. Although the analysis was systematic, there was a back-and-forth movement between the whole and parts of the text. All extracted categories and themes were reviewed and approved by the authors.

**TABLE 2:** Example of qualitative content analysis process.

Category	Subcategories	Examples of codes	Condensation	Meaning unit
Omitting entertainment and fun activities	Canceling recreational gatherings	Canceling of gatherings and recreational plans increasing conflict	Due to the cancelation of gatherings and recreational plans, conflicts among the personnel increased	”We used to go on group trips or gather around in the ward; these are all gone, and this has led to an increase in conflicts among the personnel.” (P3)
	Being away from the family for prolonged periods	Staying away from the family increasing tension and violence	Due to the not being able to relieve workplace stress, violence among the personnel increased	”I have not seen my family for months; being with family strengthens our mental status and decreases workplace tension; we are exhausted and burnt out; our mistakes have increased, and this has led to an increase in violence.” (P9)

### Trustworthiness

Guba and Lincoln’s criteria were used to determine the trustworthiness of the data (28). Credibility was confirmed by the researcher’s prolonged engagement with data, maximum variety in participants, and member and peer checks. The participants reviewed a short report of the analyzed data (member check) to see how it reflected their experiences and attitudes. Moreover, the confirmability of data was approved by two researchers (peer check) by assessing agreement on codes and themes and reviewing the text, codes, and extracted categories of the interviews while observing researcher neutrality. Data transferability was ensured through a comprehensive explanation of the data, including data collection, data analysis, direct quotations, and examples, which improved the generalizability of the findings.

### Findings

**FIGURE 1:**
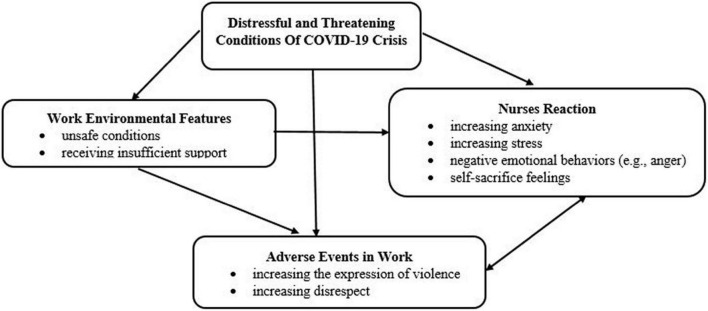
Process of the influential event on intensifying workplace violence in the COVID-19 crisis.

Analysis of the findings led to the concept of “self-sacrifice in a distressful and threatening environment: The consequences of the COVID-19 crisis in intensifying workplace violence.” There are 17 primary categories, four subcategories, and one main category (Table 3). After the continuous comparative analysis, condensation, and integration of the codes, 350 codes remained. Figure 1 presents how the affective event intensified workplace violence in the COVID-19 crisis.

**TABLE 3:** The category and subcategory related to the experiences of nurses of workplace violence in COVID.

Theme	Categories	Subcategories
Self-sacrifice in a distressful and threatening environment	Omitting entertainment and fun activities	- Canceling of recreational gatherings - Being away from the family for extended periods
	Having challenging duties in unsafe conditions	- Working with insufficient or low-quality protective equipment - Working under unfavorable physical conditions - Working in unsafe and non-standard environments - Working outside nurses’ job description - Inconsistency between duties and nurses’ abilities - Stressful and tense working conditions
	Receiving insufficient support	- Not understanding problems and lack of commitment to solving them - A lack of psychological support through counseling - Ignoring the nurses’ self-sacrificing efforts - Discriminatory attitude toward nurses - Imposing overtime work with low wages - A lack of legal support
	Nurses’ toleration of disrespect	- Visiting prohibition - Patient death, and the feeling of nurses not doing enough - Expressing stress through aggression

### Main Category: Self-Sacrifice in a Distressful and Threatening Environment: The Consequences of the COVID-19 Crisis in Intensifying Workplace Violence

According to the participants’ experiences, the impact of the COVID-19 crisis on intensifying workplace violence includes four subcategories: “omitting entertainment and fun activities,” “having challenging duties in unsafe conditions,” “receiving insufficient support,” and “nurses’ toleration of disrespect.”

#### Omitting Entertainment and Fun Activities

The participants’ experiences revealed that omitting entertainment and fun activities was one of the causes of an increase in violence among colleagues during the COVID-19 crisis. Nurses fear being infected and are afraid of transmitting the virus to others; they are away from the family for prolonged periods, which leads to high stress. Also, nurses are frequently exposed to dying patients and incurable diseases, which is an important cause of occupational stress and leads to negative emotional behaviors such as violence among nurses. In this regard, three subcategories were extracted:

##### Canceling Recreational Gatherings

The participants’ experiences revealed that as workplace stress was not relieved due to the cancelation of gatherings and recreational plans, occupational stress increased; moreover, this led to increased conflicts due to a decrease in intimacy among the personnel, leading to workplace violence. In this regard, two subcategories were extracted.

*“We used to go on group trips or gather around in the ward; these are all gone, and this has led to an increase in conflicts among the personnel.” (P3)*

##### Being Away From the Family for Prolonged Periods

The participants’ experiences revealed that being forced to stay away from the family, not being able to relieve workplace stress, and working harder due to the intense situation resulting from the COVID-19 crisis led to mental fatigue and disorders in nurses’ clinical function, leading to workplace violence.

*“I have not seen my family for months; being with family strengthens our mental status and decreases workplace tension; we are exhausted and burnt out; our mistakes have increased, and this has led to an increase in violence.” (P9)*

#### Having Challenging Duties in Unsafe Conditions

The participants’ experiences revealed that although nurses need more management support in stressful conditions, such as the COVID-19 crisis, they face unreasonable expectations and harassment from their managers. Nurses were forced to work in non-standard conditions that harmed their health during the COVID-19 crisis. For example, lack of proper ventilation and placing patients infected with COVID-19 and non-infected patients in the same rooms result in anxiety and fear related to the transmission of the COVID-19 disease and increase the aggression of the companions. Also, the heavy workload, long working hours, and inconsistency between duties and nurses’ abilities lead to burnout, emotional exhaustion, and increased interpersonal workplace conflicts between colleagues. In this regard, six subcategories were extracted.

##### Working With Insufficient or Low-Quality Protective Equipment

The participants’ experiences revealed that managers endangered the personnel’s lives by forcing them to work in unsafe conditions.

*“At the beginning of the outbreak, there were not enough scrubs, N95 face masks, and shields, and when we objected, they said that we had to accept the circumstances.” (P14)*

##### Working Under Unfavorable Physical Conditions

The participants’ experiences showed that one form of the managers’ violence was forcing nurses to work despite their poor physical condition due to COVID-19 infection; this leads to failure in providing adequate care, disorders in nurses’ clinical function, and an increase in the prevalence of mistakes, which may be the cause of increased violence against them.

*“Even if nurses’ physical conditions were not good, they were forced to come to work. For example, I took care of patients using volume expanders, and I could not finish all the work, and the nurse in charge yelled at me.” (P18)*

##### Working in Unsafe and Non-standard Environments

The participants’ experiences showed that forcing nurses to work in unsafe and non-standard environments without considering occupational safety was one form of harassment by managers.

*“The ward we were transferred to was not standard in terms of ventilation and equipment; staff health did not matter, and our objections were pointless as if our lives did not matter.” (P15)*

##### Working Outside Nurses’ Job Description

The participants’ experiences revealed that managers imposed their duties on nurses without paying attention to their heavy workload.

*“In CPR emergencies, all the follow-ups must be done by the supervisor, but they forced us to do things that were not our responsibility in such a critical situation; our objection was pointless.” (P7)*

##### Inconsistency Between Duties and Nurses’ Abilities

The participants’ experiences showed that nurses with little experience were sent to COVID-19 wards with patients who require different levels of care, leading to failure in providing adequate care and following the patient treatment plan, which may be a cause of violence against the nurses.

*“I have not had the experience of taking care of patients in critical conditions. Once, one of my patients was in bad condition, and I could not take care of my other patients. I was reprimanded for this.” (P8*)

##### Stressful and Tense Working Conditions

The participants’ experiences showed that nurses with less experience suffered from more anxiety and stress in critical situations due to insufficient knowledge and skill. Insufficient knowledge and skill lead to a decrease in concentration and an increase in the prevalence of mistakes and provide excuses for violence against nurses. Also, increased stress and tension due to patients’ bad conditions, heavy workload, and the unknown nature of the COVID-19 led to nurses’ irritability and increased interpersonal conflicts between colleagues in the workplace.

*“The workload was exhausting, and my patient was coded; I mistakenly opened the wrong medication container during CPR, and my superior yelled at me. This happens a lot.” (P16)*

#### Receiving Insufficient Support

The participants’ experiences revealed that managers’ failure to pay attention to and solve nurses’ problems, lack of solidarity and empathy, and discrimination and injustice are examples of the causes of violent behavior of managers in the workplace. On the other hand, insufficient training for nurses, failure to provide counseling services to reduce nurses’ stress and anxiety in the COVID-19 crisis, and the lack of legal protection for maltreated nurses were factors that intensified workplace violence against nurses. In this regard, six subcategories were extracted.

##### Not Understanding Problems and Lack of Commitment to Solving Them

The participants’ experiences showed that a lack of managers’ commitment to solving issues related to nurses could lead to violence against nurses.

*“We had two isolation rooms, and both were full. The supervisor called and said they were sending a COVID-19 patient. We objected, but the supervisor sent the patient anyway. Because of this, a companion of a non-COVID-19 patient became agitated and abusive.” (P1)*

##### A Lack of Psychological Support Through Counseling

The participants’ experiences revealed that ignoring counseling services during the COVID-19 crisis has led to depression, resulting in negative emotional behaviors such as violence among nurses.

*“We were suffering from depression; we were upset and had sleep disorders, and we got angry quickly. We told the authorities to bring in a psychologist to talk to the personnel, but they did not do it due to financial issues.” (P2)*

##### Ignoring the Nurses’ Self-Sacrificing Efforts

The participants’ experiences revealed that managers disregarded the nurses’ outstanding performance and self-sacrificing efforts; their selfless efforts were not appreciated, and they were even subjected to violence for minor issues.

*“It’s been several months that we are working under increased pressure; we sacrificed our lives, and we were responsible for taking care of patients whose families may have been afraid to take care of them. We need to be encouraged; if they do not appreciate us, at least they can ignore our 10- or 15-minute delays; they do not see the things we do and reprimand us for delays.” (P25)*

##### Discriminatory Attitude Toward the Nurses

The participants’ experiences revealed that managers did not treat the personnel equally and had a discriminatory and unjust attitude.

*“When the ward was less crowded, they gave off days mostly to senior nurses while we were forced to come to work, even when we were sick.” (P10)*

##### Imposing Overtime Work With Low Wages

The participants’ experiences demonstrated that although the nurses had made much self-sacrificing effort during the COVID-19 crisis and had done everything in their power, they did not receive any financial benefits to stay motivated.

*“Our payment is quite low, and they have even further decreased our payment even though our work has not decreased but even increased due to the COVID-19 crisis. I am working by patients*’ *bedsides day and night. We cannot do anything about this injustice.” (P24)*

##### Lack of Legal Support

The participants’ experiences showed that managers did not provide legal support for maltreated nurses, and this facilitated workplace violence against nurses.

*“The authorities do not do anything against aggressive companions; unfortunately, the patients’ rights charter has several articles, but the nurses’ rights chart has only one.” (P22)*

#### Nurses’ Toleration of Disrespect

The participants’ experiences revealed that nurses were subjected to violence by patients and their companions for unfair reasons; moreover, the stressful circumstances of the COVID-19 crisis led to irritability and aggression in the personnel, especially physicians. In this regard, three subcategories were extracted.

##### Visiting Prohibition

The participants’ experiences revealed that companions did not understand the limitations of visiting COVID-19 patients and directed their anger toward nurses.

*“Due to the COVID-19 crisis, we tell companions that for their safety, visiting is prohibited; however, patients*’ *companions get upset and insult us.” (P21)*

##### Patient Death and the Feeling of Nurses Not Doing Enough

The participants’ experiences showed that companions accuse nurses of irresponsibility and subject them to violence because they cannot cope with the death of their loved ones. Nurses who had done their best to care for the patients felt that they were subjected to disrespect and violent treatment unfairly.

*“During this time, we have experienced different types of violence; even though we were exhausted and weak and we did our job the best that we could, our tireless efforts were ignored. For instance, when I gave the news of the death of a patient to their companions, they grabbed me and pushed me with force and punched and kicked me.” (P19)*

##### Expressing Stress Through Aggression

The participants’ experiences revealed that the stressful circumstances of the COVID-19 crisis and the unknown nature of the disease led to the irritability of the personnel, especially the physicians, and they expressed this stress and anxiety through aggression toward nurses.

*“One of the physicians was unable to intubate a COVID-19 patient and threw the tracheal tube at us angrily without any reason, not considering that we did not have any glasses or shields; We cannot do anything about this violent behavior, and our objections are pointless.” (P13)*

## Discussion

This qualitative study aimed to study Iranian nurses’ experiences of workplace violence during the COVID-19 crisis. This study emphasizes the need to design educational programs and prevention strategies to manage the destructive psychological and occupational effects of the crisis on nurses. The current study revealed that despite the nurses’ good performance and selfless efforts, their hard work was ignored, and they were subjected to disrespect and violent treatment. Despite numerous studies in Iran on the impact of the COVID-19 crisis on nurses, this is the first qualitative study about how the COVID-19 crisis has intensified workplace violence. The analysis of nurses’ experiences revealed: four subcategories: “omitting entertainment and fun activities,” “having challenging duties in unsafe conditions,” “receiving insufficient support,” and “nurses’ toleration of disrespect.”

### Omitting Entertainment and Fun Activities

Nurses experienced stressful lifestyles and work conditions due to the COVID-19 pandemic, which increased irritability and anger due to the lack of opportunities and services to help reduce stress and depression. Studies conducted in several hospitals in Iran showed that most nurses in COVID-19 wards had high levels of anxiety and depression (29). Additionally, the results of a systematic review revealed that workplace violence has a positive relationship with anxiety and burnout (30). According to a study, there is a relationship between anger and symptoms of depression; moreover, stress causes symptoms of anxiety and depression, and these factors are influential in causing anger (31). Similarly, Magnavita et al. (32) discovered a relationship between workplace violence and symptoms of anxiety (32). It can be stated that nurses’ failure to relieve workplace stress and the increased workplace’ tension caused by the special conditions of the COVID-19 pandemic result in more anxiety and depression; these factors are barriers to anger management and they intensify violence among nurses.

### Having Challenging Duties in Unsafe Conditions

During the COVID-19 pandemic, nurses were harassed by managers who forced them to do extra work in non-standard work environments and unfavorable physical conditions. Some studies have shown that nurses experienced a lot of stress and anxiety during the pandemic due to limited access to personal protective equipment (2). The results of a study conducted in Iran on the experience of emergency nurses in the COVID-19 pandemic indicated that most hospitals are far from ideal: Patient care is sometimes inadequate, nurses’ experience is limited, the number of nurses is low, support services are inadequate, and good quality equipment is often unavailable (33). According to studies, heavy workload, unsafe work environments, limited resources, patient care problems, and conflicts with colleagues and supervisors can become constant sources of stress in nurses’ workplaces (34). Increased work-related stress and anxiety will potentially increase violence (35, 36). Not receiving enough support in spite of the heavy workload and their demanding duties has worsened nurses’ emotional and mental health, which can increase the violence they are subjected to (14). Nurses suffer from high levels of stress due to working in unsafe conditions, which, coupled with inadequate support from managers, makes them vulnerable to physical and emotional exhaustion. Managers expect nurses to be committed to their duties, even by resorting to violence toward the nurses. Nurses who had experienced long-term stress due to forced work in unsafe conditions and perceived violent behavior were at risk of depression and anxiety. In addition to physical and emotional burnout, these factors could provide bases for nurses’ irritability, aggression, and violent behaviors to each other.

### Receiving Insufficient Support

The non-supportive work environment increased violence against nurses. Hospitals face many crises; nurses are constantly experiencing these crises and have to cope with them. Poor crisis management leads to more crises and imposes extra workload on the nurses (33), with the increased workload increasing the violence in the workplace (36). Also, studies have shown the positive effect of psychological counseling in reducing occupational stress (34). In this regard, a study has emphasized the necessity of attention to health promotion programs and preventive intervention in the workplace, considering the significant psychological impact the COVID-19 crisis has had on healthcare workers; this study recommended that policy-makers plan interventions, including supplying pertinent information and psychosocial support, acknowledging success, increasing resilience, and monitoring the health status of staff (37). Studies have also shown that the culture of self-sacrifice leads to increased physical and emotional exhaustion in the nurse’s workplace (3). According to studies, occupational stress, whether accompanied by workplace violence or not, increases the risk of harm associated with facing violence (32). Also, a study has shown that the COVID-19 crisis highlighted the existing structural problems in health management. Programs for the COVID-19 crisis management were either not completely developed or not correctly implemented. Tension, complaints, and despair can lead to conflict in healthcare settings. Lack of coordination between managers and policy-makers escalates conflicts, and the resulting complaints are not addressed (38). Managers did not have the necessary preparation for the crisis, and they forced nurses to work in these conditions without proper facilities and support, even by resorting to violence toward the nurses. They expected nurses who work selflessly to be committed to their patients and profession. Nurses experienced stress because they were unprepared for the crisis; they were forced to work in these circumstances and experienced injustice and violent behavior. Furthermore, providing optimal care and self-sacrifice leads to mental and physical fatigue in nurses. Thus, chronic workplace stress, extreme fatigue, and burnout could increase the risk of workplace violence against nurses.

### Nurses’ Toleration of Disrespect

Although nurses worked selflessly and did their best to take care of the patients, they were subjected to disrespect and violence by the patients’ companions. Also, the tense circumstances increased irritability in the personnel, especially physicians, and led to violence against nurses (15). Also, according to research, misunderstandings and high levels of anxiety and stress in patients and their companions during emergencies and poor stress management increase violence against nurses (15). In addition, nurses provide 24-h direct care for the patients; prolonged exposure makes them more likely to experience patients’ and their companions’ aggressive behavior (10). Furthermore, in the COVID-19 crisis, because health care providers were under high job pressure, they had very limited time to interact with the patient’s relatives. Limited interaction with relatives increases the risk of misunderstandings and the danger of negligence litigation, so when they had to report the failure of treatment to the families, the risk of violent reaction was higher (38). Some people have problems in anger management, and when they lose a loved one, they do not know how to cope with it; because people are not trained for this and nurses are the first healthcare providers that they meet in hospitals, they direct their aggression toward nurses. People’s inadequate awareness of the nature of COVID-19 and unawareness of the nurses’ efforts were other reasons for violence against nurses. Also, the harsh situation and poor stress and anxiety management in health care providers, especially in physicians, decreased their tolerance; these healthcare providers subjected the other personnel, especially nurses, to their aggression as a negative coping strategy for adapting to this stress.

### Limitations

The limitation of this study is that it may not be generalizable to all cultures because experiencing workplace violence may be affected by cultural and social factors. Therefore, we recommend that similar studies be conducted in other societies.

## Conclusion

In conclusion, the COVID-19 crisis caused distressful and threatening conditions aggravated by unsafe work conditions and insufficient support from managers, leading to intensified workplace violence against nurses; despite the nurses’ self-sacrificing efforts, they were subjected to them to disrespect and violence. It is recommended that managers and health policy-makers consider the long-term psychological consequences of COVID-19 as a significant public health problem, especially regarding the most vulnerable personnel. Providing educational courses for crisis management and stress and anger management helps nurses deal with such problems.

## Data Availability Statement

The raw data supporting the conclusions of this article will be made available by the authors, without undue reservation.

## Ethics Statement

The studies involving human participants were reviewed and approved by IR.KMU.REC.1398.174. The patients/participants provided their written informed consent to participate in this study.

## Author Contributions

PM, AR, and FA: study conception and design. PM and ZE: data collection. All authors: data analysis and interpretation, drafting of the article, and critical revision of the article.

## Conflict of Interest

The authors declare that the research was conducted in the absence of any commercial or financial relationships that could be construed as a potential conflict of interest.

## Publisher’s Note

All claims expressed in this article are solely those of the authors and do not necessarily represent those of their affiliated organizations, or those of the publisher, the editors and the reviewers. Any product that may be evaluated in this article, or claim that may be made by its manufacturer, is not guaranteed or endorsed by the publisher.
