# Supplementary material for: Self-Sacrifice in a Distressful and Threatening Environment: The Consequences of the COVID-19 Crisis in Intensifying Workplace Violence
Source: Front Psychiatry. 2022 May 17;13:848059. doi: 10.3389/fpsyt.2022.848059 (PMC9157344; doi:10.3389/fpsyt.2022.848059)
Supplement: Supplementary file 1 [file Table_1.DOCX]

**Consolidated criteria for reporting qualitative studies (COREQ): 32-item checklist**

| **No.** | **Item** | **Description** | **Section #** |
| --- | --- | --- | --- |
| **Domain 1: Research team and reflexivity** | | |  |
| Personal characteristics | | |  |
| *1.* | Interviewer/facilitator | Which author/s conducted the interview or focus group? | First author |
| *2.* | Credentials | What were the researcher's credentials? *E.g., PhD, MD* | PhD |
| *3.* | Occupation | What was their occupation at the time of the study? | PhD candidate |
| *4.* | Gender | Was the researcher male or female? | Female |
| *5.* | Experience and training | What experience or training did the researcher have? | I have passed many qualitative research courses, and I had written this qualitative article under the supervision of my professors |
| Relationship with participants | | |  |
| *6.* | Relationship established | Was a relationship established prior to study commencement? | Yes. I had a history of clinical work in the hospital, understood the nurses' problems and issues, and established a close relationship with the participants. |
| *7.* | Participant knowledge of the interviewer | What did the participants know about the researcher? *E.g. Personal goals, reasons for doing the research* | Firstly, I introduced myself to my participants and obtained informed written consent for data recording and assuring participants of the confidentiality of data. Also, before doing the interviews, I provided some information, including study aims and collection method, to the participants. |
| *8.* | Interviewer characteristics | What characteristics were reported about the interviewer/facilitator? *E.g. Bias, assumptions, reasons and interests in the research topic* | Reasons and interests in the research topic |
| **Domain 2: Study design** | | |  |
| Theoretical framework | | |  |
| *9.* | Methodological orientation and theory | What methodological orientation was stated to underpin the study? *E.g. grounded theory, discourse analysis, ethnography, phenomenology, content analysis* | content analysis |
| Participant selection | | |  |
| *10.* | Sampling | How were participants selected? *E.g. purposive, convenience, consecutive, snowball* | Sampling was done through purposeful sampling |
| *11.* | Method of approach | How were participants approached? *E.g. face to-face, telephone, mail, email* | Using individual, face-to-face, in-depth interviews |
| *12.* | Sample size | How many participants were in the study? | In the present study, saturation was reached after interviewing 20 participants |
| *13.* | Non-participation | How many people refused to participate or dropped out? What were the reasons for this? | I had not any drop. |
| Setting | | |  |
| *14.* | Setting of data collection | Where was the data collected? *E.g. home, clinic, workplace* | The study setting included referral hospitals of X. |
| *15.* | Presence of nonparticipants | Was anyone else present besides the participants and researchers? | NO |
| *16.* | Description of sample | What are the important characteristics of the sample? *E.g. demographic data, date* | Based on the maximum variation, nurses were different in terms of age, gender, work experience, work experience in COVID-19 wards, and working at fixed and rotating shifts. Personal information such as marital status, academic degree, and position was recorded to provide a wide range of information |
| Data collection | | | |
| *17.* | Interview guide | Were questions, prompts, guides provided by the authors? Was it pilot tested? | The participants were individually interviewed by first author according to the interview guide |
| *18.* | Repeat interviews | Were repeat interviews carried out? If yes, how many? | No |
| *19.* | Audio/visual recording | Did the research use audio or visual recording to collect the data? | All interviews were recorded |
| *20.* | Field notes | Were field notes made during and/or after the interview or focus group? | Yes. Field notes were used for data collecting, in addition to the above-mentioned semi-structured in-depth interviews |
| *21.* | Duration | What was the duration of the interviews or focus group? | The interviews lasted from 45 to 90 minutes. |
| *22.* | Data saturation | Was data saturation discussed? | Yes |
| *23.* | Transcripts returned | Were transcripts returned to participants for comment and/or correction? | Yes |
| **Domain 3: analysis and findings** | | | |
| Data analysis | | | |
| *24.* | Number of data coders | How many data coders coded the data? | After constant comparative analysis, condensation, and integration of the codes, 350 codes resulted. |
| *25.* | Description of the coding tree | Did authors provide a description of the coding tree? | Yes |
| *26.* | Derivation of themes | Were themes identified in advance or derived from the data? | Yes |
| *27.* | Software | What software, if applicable, was used to manage the data? | MAXQDA 10 was used in order to code and extract categories and themes |
| *28.* | Participant checking | Did participants provide feedback on the findings? | The participant reviewed a short report of the analyzed data for member check to see how well the report reflected their experiences and attitudes |
| Reporting | | | |
| *29.* | Quotations presented | Were participant quotations presented to illustrate the themes / findings? Was each quotation identified? *E.g. Participant number* | Yes |
| *30.* | Data and findings consistent | Was there consistency between the data presented and the findings? | Yes |
| *31.* | Clarity of major themes | Were major themes clearly presented in the findings? | Yes |
| *32.* | Clarity of minor themes | Is there a description of diverse cases or discussion of minor themes? | Yes |
